# P17 A case of treatment resistant severe destructive arthritis in a young boy

**DOI:** 10.1093/rap/rkab068.016

**Published:** 2021-10-19

**Authors:** Lucy Paterson-Brown, Alexandra Jones, Nick Wilkinson

**Affiliations:** Evelina London Children's Hospital, London, United Kingdom

## Abstract

**Case report - Introduction:**

Major advances in medical and therapy management of inflammatory arthritis has been associated with marked improvement in outcomes. We present a very unusual case of a young boy with destructive and incapacitating arthritis of knees, feet and ankles refractory to such management. He is now wheelchair dependent. We have evidence of reversal of bone loss after treatment. However, synovitis and joint destruction has left few surgical options with above knee amputation considered to have the best long-term functional outcome.

**Case report - Case description:**

Age five, he had persistent pain and swelling of the right knee after a minor fall. In the emergency department a sclerotic, lucent area was highlighted on X-ray. Neoplasia was ruled out and the changes were felt to be a result of erosive arthropathy.

By the time of rheumatology referral, symptoms progressed to significant bilateral knee effusions with left ankle inflammation. All three joints underwent steroid injections which had a good response, but unfortunately this was short-lived and they were repeated. A synovial biopsy was sent to exclude other pathologies and loose bone fragments were removed from both knees. Biopsy confirmed chronic inflammation and methotrexate was commenced. Unfortunately, there was minimal response.

Over the next 5 years he trialled etanercept, infliximab, tocilizumab, pamidronate, azathioprine and abatacept. His destructive arthritis of the knees, ankles and feet continued to progress with relentless bone loss and associated osteochondritis dissecans (OCD).

Opinions from five other tertiary rheumatology units worldwide, three radiology departments and four orthopaedic units were sought. Unable to control the synovitis and in the presence of complete loss of the lateral femoral condyle we elected to refocus our attention to bone integrity and preparation for definitive surgery. This included the use of denosumab. Serial radiology showed reversal of some bone loss over 2 years but surgical teams could not identify intervention to optimise weight-bearing capacity.

Mobility deteriorated to leave our patient wheelchair dependent outside, using crutches or his knees to move around inside. Further surgical consideration has indicated the best long-term will be associated with above knee amputations.

**Case report - Discussion:**

Our team are aware of one less severe case in a 13-year-old male in Oxford that stabilised after 5 years, allowing definitive treatment with bilateral knee replacement followed by excellent recovery with full mobility. None of the international centres we contacted reported a case as severe as ours. From discussions with our paediatric and adult orthopaedic colleagues, the only comparable patients they have seen this level of bony knee destruction in has been uncontrolled haemophilia patients. However, the damage is not seen at such a young age which affects the surgical options due to ongoing growth in childhood and adolescence. This caused us to question; why was our patient so severely affected? Would a different treatment course have been feasible? And would this have changed the outcome?

Despite trial of many immunomodulatory agents, both those routinely used for arthritis and those used in treatment resistance, the persistent inflammation and destruction progressed. As a result he developed persistent and problematic OCD. OCD is considered a relatively common diagnosis that can occur in adults and children of unclear aetiology. A small subchondral bone fragment becomes loose in the joint space as a result of disrupted blood supply. OCD seems to be more common in males and is often managed conservatively. In children, the knee is the most commonly affected joint and inflammation is hypothesised as a possible cause which we will elaborate on in our presentation. For cases requiring surgical intervention this is normally localised drilling, fixation or bone grafts; however, most cases only affect one site, with one loose fragment which was very different from our patient.

**Case report - Key learning points:**

From this case we have been reminded that each patient follows a different clinical course and response to medications is difficult to predict. We have gained experience managing difficult inflammatory arthritis-related OCD working closely with our orthopaedic colleagues. Although we managed to control the destructive inflammatory process this did not reverse the damage to the patient’s joints and we have been left with no choice but to progress to surgical treatment options.

By sharing this case we hope to learn from any similar experiences or challenges faced by other clinical teams to help inform our practice for any other cases in the future.

